# circRNA_101277 Influences Cisplatin Resistance of Colorectal Cancer Cells by Modulating the miR-370/IL-6 Axis

**DOI:** 10.1155/2022/4237327

**Published:** 2022-03-14

**Authors:** Qing Lv, Qinghua Xia, Anshu Li, Zhiyong Wang

**Affiliations:** Gastrointestinal Surgery, Wuhan Union Hospital, Wuhan, China

## Abstract

**Background:**

Colorectal cancer (CRC) is among the most prevalent malignancies globally. Early detection of precancerous lesions through routine colonoscopy has led to a dramatic reduction in CRC-related incidence and mortality among those between the ages of 50 and 70. However, in those where the disease progresses to an advanced stage, chemotherapy remains the primary available treatment option, and the associated 5-year survival rate remains low. The identification of genes associated with CRC chemoresistance would thus be a beneficial approach to identifying novel treatments for this deadly disease.

**Methods:**

The expression of circRNA_101277, miR-370, and IL-6 was assessed via qRT-PCR. IL-6 levels were measured with a human IL-6 ELISA kit based on the provided protocols. CRC cellular proliferation and cisplatin IC50 values were quantified via MTT assays. Luciferase assays were used to detect circRNA_101277 and miR-370 binding sites or miR-370 and IL-6 binding sites.

**Results:**

circRNA_101277 was increased in CRC tissues compared with control samples. circRNA_101277 overexpression was evident in CRC cells, and knockdown of this circRNA suppressed cellular proliferation and cisplatin resistance in these cancer cells. At a mechanistic level, circRNA_101277 was found to function by sequestering miR-370, thereby upregulating the miR-370 target gene IL-6 and promoting cisplatin resistance via this miR-370/IL-6 axis.

**Conclusion:**

In summary, our data highlight circRNA_101277 as a novel driver of CRC cell cisplatin resistance that functions by sequestering miR-370 and thereby enhancing IL-6 expression. These findings suggest that this circRNA_101277/miR-370/IL-6 axis may represent a critical axis of chemoresistance in CRC that can be targeted to diagnose and/or treat this cancer.

## 1. Introduction

Colorectal cancer (CRC) is among the most prevalent malignancies globally [1–4]. Early detection of precancerous lesions through routine colonoscopy has led to a dramatic reduction in CRC-related incidence and mortality among those between the ages of 50 and 70 [5]. However, in those where the disease progresses to an advanced stage, chemotherapy remains the primary available treatment option, and the associated 5-year survival rate remains low. The identification of genes that contribute to the therapeutic resistance in CRC tumors thus represents an essential approach to improving treatment options for those patients afflicted by this cancer type.

Circular RNAs (circRNAs) are ring-shaped RNA molecules formed by 3′ and 5′ end-joining [6–8]. The expression of many circRNAs is dysregulated in tumors, and as such, these circRNA expression patterns can be utilized for diagnostic purposes or as potential therapeutic targets. At a functional level, these circRNAs can also function as competing endogenous RNAs that bind to and sequester specific microRNAs (miRNAs), thus preventing them from interacting with their target mRNA molecules. For example, circ_0020710 is a circRNA which modulates the miR-370-3p/CXCL12 axis in melanoma and thereby mediates cancer progression and immune evasion [9]. In a similar manner, circBANP can sequester let-7a, thereby driving gastric cancer progression via the FZD5/Wnt/*β*-catenin pathway [10]. Prior work has further shown that circNRIP1 drives cervical cancer cell invasion and migration via binding to miR-629-3p and controlling the PTP4A1/ERK1/2 axis [11].

Herein, we identified circRNA_101277 as a novel circRNA that is upregulated in CRC tumor tissues and that is positively correlated with the proliferation of CRC cells. We additionally determined that circRNA_101277 controls CRC cell cisplatin resistance via modulating the miR-370/IL-6 axis.

## 2. Materials and Methods

### 2.1. Patients

100 CRC tissue samples and surrounding healthy tissue samples were collected from individuals who had not undergone neoadjuvant therapy at our hospital from January 2015–December 2019. The Ethics Committee in our hospital, Wuhan Union Hospital, approved this study, and all patients or their family members provided written informed consent.

### 2.2. Cell Culture

Human colonic epithelial cells (CCD 841 CoN) and colorectal cell lines (LoVo, HT-29, HCT116, SW480, and SW620) were obtained from the Cell Bank of the Type Culture Collection (Shanghai City, China) and were grown in RPMI-1640 (Gibco, Thermo Fisher Scientific, China) containing 10% FBS (Gibco) and penicillin/streptomycin in a 5% CO2 humidified incubator at 37°C.

### 2.3. qRT-PCR

TRIzol (Invitrogen) was used to isolate total RNA from tissue and cell samples based on provided directions, after which a NanoDrop ND2000 spectrophotometer (NanoDrop) was used to evaluate the yield and quality of each RNA sample. Reaction mixtures (20 µL) that contained 1 µg of total RNA were used to generate cDNA with PrimeScript RT‐polymerase (Takara), after which SYBR Green Premix Ex Taq (Takara Bio) was used to conduct qRT-PCR reactions with an ABI PRISM 7500 Sequence Detection System (Applied Biosystems, Life Technologies). All gene expression was normalized to GAPDH. Primers used for this study were as follows: circRNA_101277 forward: 5′- GCCCAGCTTTTCCATCCTGG-3′, reverse: 5′-TCATCCGCTCCTCTGGCATCATAG-3′; TERT forward: 5′-GTCATCGCCAGCATCATC-3′, reverse: 5′- CTGTTCACAAGCTTTCAAGAAAACG-3′; IL-6 forward: 5′-GGCCCTTGCTTTCTCTTCG-3′, reverse: 5′-ATAATAAAGTTTTGATTATG-3′; GAPDH forward: 5′-GGGAAACTGTGGCGTGAT-3′, reverse: 5′-GAGTGGGTGTCGCTGTTGA-3′.

### 2.4. MTT Assay

CRC cell proliferation and cisplatin IC50 values were assessed via the MTT assay based on provided directions, as detailed in prior studies [12].

### 2.5. Luciferase Assay

Luciferase assays were conducted as discussed previously [13]. In brief, luciferase reporter vectors were transfected into HCT116 and SW620 cells. At 24 h posttransfection, cells were collected, and luciferase activity was assessed with a Dual‐Luciferase Reporter Assay System (Promega) based on the provided directions.

### 2.6. ELISA

IL-6 levels were measured with a human IL-6 ELISA kit (Abcam) based on the provided protocols.

### 2.7. Statistical Analysis

The data are given as means ± SD and were analyzed via two-tailed Student's *t*-tests or one-way ANOVAs as appropriate. *P* < 0.05 was the significance threshold for this study.

## 3. Results

### 3.1. circRNA_101277 Is Upregulated in CRC

**Figure 1:**
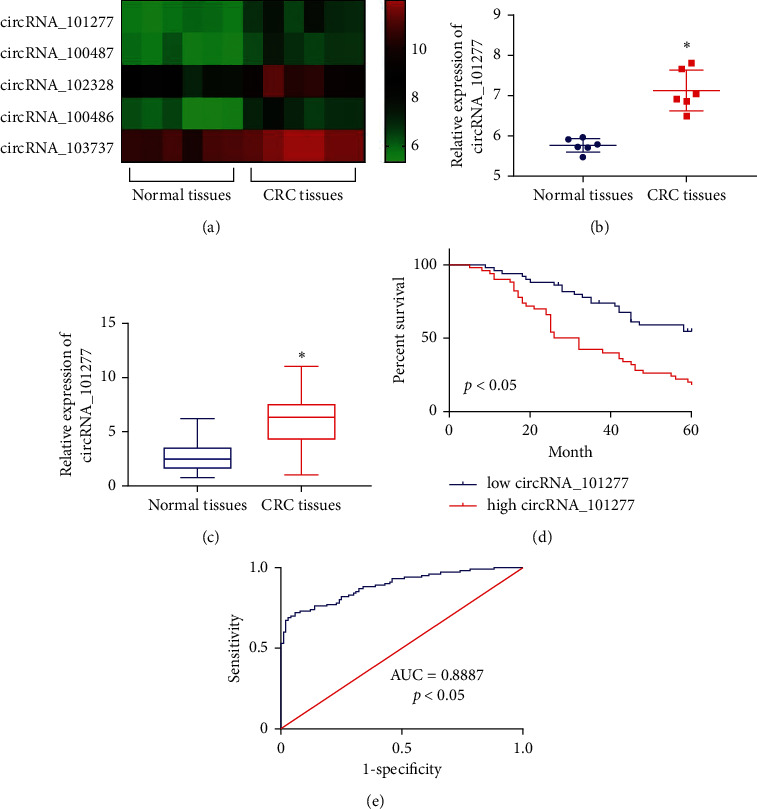
CRC is associated with circRNA_101277 upregulation. (a) Hierarchical clustering analyses were used to detect differences in circRNA expression profiles between CRC and healthy tissues. (b) Relative circRNA_101277 levels were measured in normal tissues and CRC tissues. (c) circRNA_101277 levels in 100 pairs of CRC and healthy tissues were assessed via qRT-PCR. (d) The overall survival of CRC patients with high circRNA_101277 expression was significantly worse than that of patients with low expression of this circRNA. (e) ROC curve analyses revealed that circRNA_101277 was able to effectively discriminate between tumor and adjacent tissues.  ^*∗*^*p* < 0.05.

We began by analyzing patterns of circRNA expression in 6 CRC patient samples from the GSE138589 GEO microarray dataset. This analysis revealed 250 total circRNAs that were upregulated in CRC patients, with the top 5 of these circRNAs being shown in Figure 1(a). We then selected the most differentially expressed of these circRNAs (circRNA_101277) for further analysis (Figure 1(b)). To gauge the clinical relevance of circRNA_101277, we then assessed its expression in 100 CRC tumor tissue samples and 100 control samples via qRT-PCR, confirming that this circRNA was significantly upregulated in CRC tissues relative to normal controls (Figure 1(c)). We additionally found that circRNA_101277 was associated with tumor size (*P* < 0.05), TNM stage (*p* < 0.05), and metastasis (*P* < 0.05), but not age or gender (*P* > 0.05) (Table 1). Kaplan–Meier survival curves further revealed that patients expressing high levels of circRNA_101277 significantly decreased overall survival relative to patients expressing low levels of this circRNA (Figure 1(d)), and ROC curve analyses revealed that circRNA_101277 could be reliably used to discriminate between tumor and adjacent tissues (Figure 1(e)).

### 3.2. circRNA_101277 Downregulation Attenuates Cisplatin Resistance in CRC

**Figure 2:**
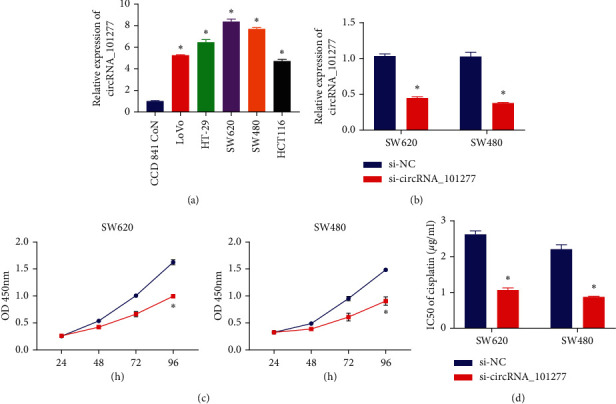
circRNA_101277 downregulation overcomes CRC cisplatin resistance. (a) circRNA_101277 levels in CRC cells were measured. (b) circRNA_101277 knockdown was achieved in SW620 and SW480 cells. (c) circRNA_101277 knockdown impaired SW620 and SW480 cellular proliferation. (d) circRNA_101277 downregulation decreased the IC50 of cisplatin in CRC cells. ^∗^*p* < 0.05.

To explore the functional relevance of circRNA_101277 as a regulator of cisplatin resistance, we next quantified the expression of this circRNA in a range of CRC cell lines (Figure 2(a)). We then knocked down circRNA_101277 in SW620 and SW480 cells which expressed higher levels of this circRNA (Figure 2(b)), and we found that such knockdown inhibited the proliferation of these cells, indicating that circRNA_101277 may be an oncogene (Figure 2(c)). In addition, circRNA_101277 knockdown decreased the cisplatin IC50 value in CRC cells, thus indicating that this circRNA increases CRC cell chemoresistance (Figure 2(d)).

### 3.3. circRNA_101277 Sequesters miR‐370

**Figure 3:**
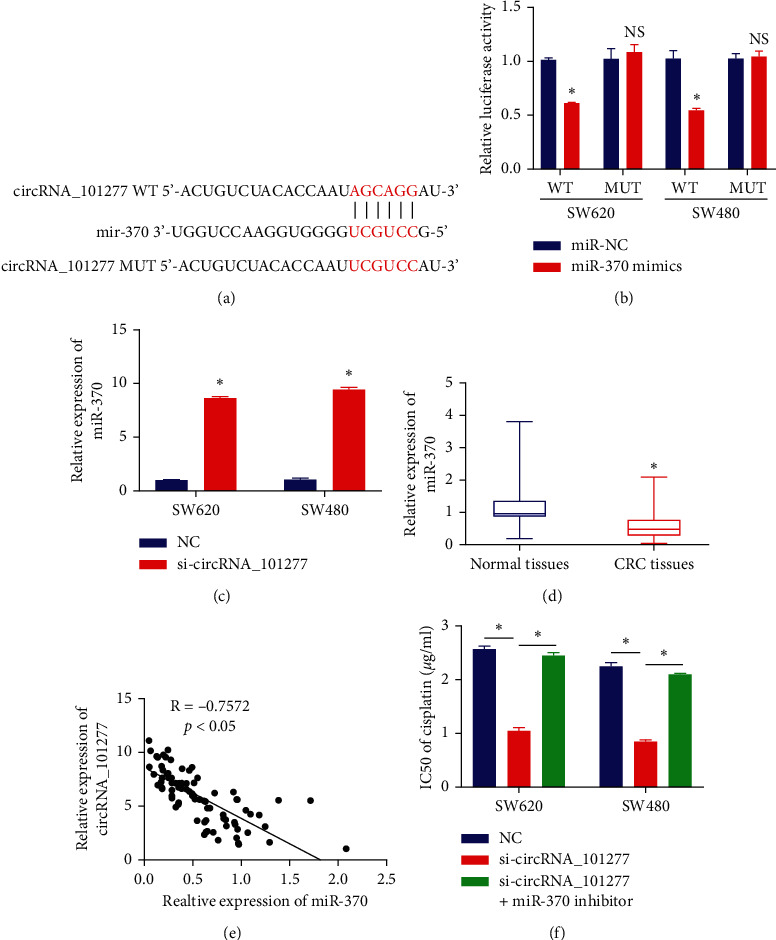
circRNA_101277 sequesters miR‐370. (a) Putative miR-370 binding sites within circRNA_101277. (b) Relative circRNA_101277‐WT luciferase activity was decreased by miR‐370 mimics in CRC cells. (c) qRT-PCR was used to assess miR-370 expression. (d) CRC tissues exhibited marked miR-370 downregulation relative to normal tissues. (e) miR-370 expression was negatively correlated with that of circRNA_101277 in CRC tissues. (f) An MTT assay was used to assess the cisplatin IC50 value in the indicated cells. ^∗^*p* < 0.05.

To understand how circRNA_101277 influences CRC cell proliferation and chemoresistance, we next assessed putative targets for this circRNA with the miRBase and circInteractome databases, which revealed miR-370 as one such potential target (Figure 3(a)). Luciferase assays revealed that miR-370 mimics were sufficient to decrease circRNA_101277‐WT reporter activity in CRC cells (Figure 3(b)), and miR-370 expression was significantly elevated in SW620 and SW480 cells following circRNA_101277 knockdown (Figure 3(c)). In contrast, miR-370 expression in CRC tissues was decreased relative to levels in normal tissues (Figure 3(d)), and in CRC tissues the expression of this miRNA was negatively correlated with that of circRNA_101277 (Figure 3(e)). Importantly, transfection of these circRNA_101277-knockdown cells with a miR-370 inhibitor was sufficient to restore cisplatin IC50 values (Figure 3(f)), indicating that circRNA_101277 bolsters cisplatin resistance at least in part by suppressing the expression of miR-370.

### 3.4. circRNA_101277 Modulates the miR-370/IL-6 Axis to Enhance CRC Cell Chemoresistance

**Figure 4:**
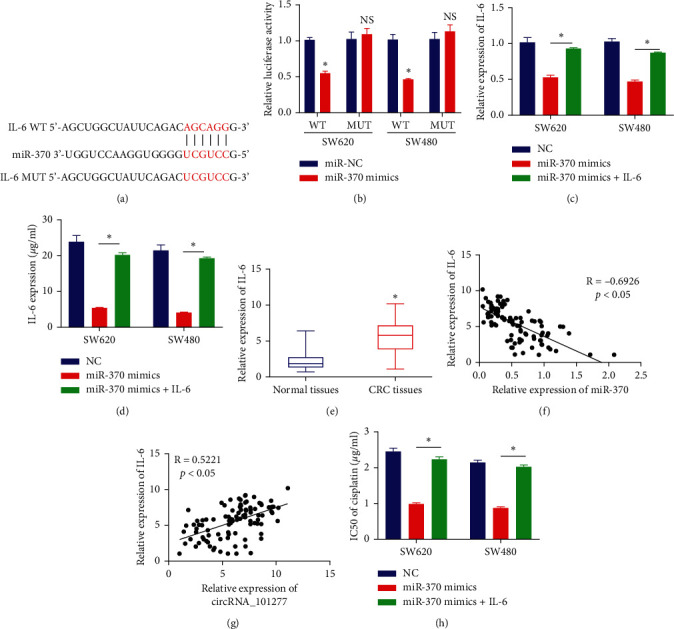
circRNA_101277 modulates the miR-370/IL-6 axis to enhance cisplatin resistance. (a) Putative miR‐370 binding sites within the IL-6 3′‐UTR. (b) miR-370 mimics decreased the luciferase activity of the IL‐6-WT reporter in CRC cells. The expression of IL-6 was measured via qRT-PCR (c) and ELISA (d). (e) IL-6 expression was markedly elevated in CRC tissues relative to healthy control tissue. (f) The expression of IL-6 in CRC tissues was negatively correlated with that of miR-370. (g) The expression of IL-6 was positively correlated with that of circRNA_101277 in CRC tissues. (h) An MTT assay was used to assess the cisplatin IC50 value in the indicated cells. ^∗^*p* < 0.05.

To understand the mechanistic basis whereby miR-370 influences CRC cell cisplatin resistance, we next leveraged the TargetScan and miRDB databases to identify IL-6 as a potential miR‐370 target gene (Figure 4(a)). Consistent with this prediction, miR-370 mimics were able to reduce the luciferase activity associated with a WT IL-6 reporter construct in CRC cells (Figure 4(b)). We further found that IL-6 expression was markedly reduced in SW620 and SW480 cells following miR-370 mimic transfection, and this expression was restored upon IL-6 overexpression (Figure 4(c) and 4(d)). CRC tissues also exhibited significantly increased IL-6 expression relative to levels in normal tissues (Figure 4(e)), and within these CRC samples the expression of miR-370 was negatively correlated with that of IL-6 (Figure 4(f)), while it was positively correlated with circRNA_101277 expression (Figure 4(g)). IL-6 transfection was also sufficient to restore cisplatin IC50 values in SW620 and SW480 cells following miR-370 mimic transfection (Figure 4(h)), indicating that circRNA_101277 augments cisplatin resistance via modulating the miR-370/IL-6 axis.

## 4. Discussion

Given that circRNAs are highly stable and are expressed in a tissue-specific fashion, they may represent ideal biomarkers for the detection and analysis of CRC. Consistent with such a model, prior reports have shown that the circ_0021977/miR‐10b‐5p/P21 and P53 axis functions to impair CRC cell proliferation, migration, and invasion [14]. Hsa_circ_0001178 similarly controls CRC metastasis and invasion by sequestering several miRNAs and thereby promoting ZEB1 upregulation [15]. The circRNA NSD2 can also promote CRC cell metastasis via targeting miR-199b-5p-mediated DDR1 and JAG1 signaling [16]. Herein, we identified circRNA_101277 as a novel CRC-related circRNA by analyzing the GSE138589 microarray dataset. This circRNA was upregulated in CRC tissues relative to normal tissues, and its expression was positively correlated with tumor size, TNM stage, metastatic progression, and patient OS. Consistent with this, we found that circRNA_101277 was able to readily differentiate between tumor and paracancerous tissues. When circRNA_101277 was knocked down in CRC cells, it suppressed their proliferation, indicating that it may serve an oncogenic role within these tumor cells. In line with prior reports that circRNAs can serve as vital regulators of tumor chemoresistance [17–19], we found that circRNA_10277 decreased cisplatin IC50 values in CRC cells, suggesting that this circRNA impaired their resistance to this common chemotherapeutic agent.

Prior studies have found miR-370 to function as a tumor suppressor in a range of cancer types. For example, in the context of oral carcinoma, miR-370 controls the expression of insulin receptor substrate-1 and suppresses tumor growth [20]. Similarly, miR-370 functions as a tumor suppressor in laryngeal squamous cell carcinoma (LSCC) by targeting FoxM1 [21]. This miRNA can further downregulate oncogenic TRAF4 to inhibit non-small-cell lung cancer (NSCLC) progression [22]. Herein, we determined that knocking down circRNA_101277 resulted in the upregulation of miR-370, and we further found that miR-370 was significantly downregulated in CRC tissues relative to normal control tissues. Importantly, miR-370 expression in CRC tissues was negatively correlated with that of circRNA_101277, suggesting that this circRNA functions at least in part by sequestering miR-370 and inhibiting its functionality. Consistent with this hypothesis, transfecting CRC cells with a miR-370 inhibitor was sufficient to normalize cisplatin IC50 values in circRNA_101277-knockdown CRC cells, suggesting that this circRNA suppresses miR-370 expression and thereby enhances cisplatin resistance.

Many recent reports have demonstrated a role for IL-6 as a driver of tumor drug resistance. In the context of hepatocellular carcinoma, for example, IL-6 promotes resistance to sorafenib and is associated with poor prognosis [23]. In NSCLC, IL-6 signaling also promotes the upregulation of DNA repair-related and antiapoptotic genes, thereby enhancing tumor cell resistance to cisplatin [24]. IL-6 is similarly associated with STAT3-mediated cisplatin resistance in breast cancer [25], and with acquired cisplatin resistance and poor prognosis in head and neck squamous cell carcinoma [26]. Herein, we determined that IL-6 was significantly upregulated in CRC tissues relative to normal controls, and we determined that such IL-6 expression was negatively correlated with miR-370 levels and positively correlated with circRNA_101277 levels within these tumor tissues. When CRC cells were transfected with miR-370 mimics, this significantly inhibited IL-6 expression, indicating that miR-370 directly suppresses IL-6 expression within CRC cells. When miR-370 mimic-transfected cells were also transfected to overexpress IL-6, this was sufficient to lower cisplatin IC50 values in these cells, thus indicating that circRNA_101277 enhances CRC cell cisplatin resistance via modulating miR-370/IL-6 axis.

## 5. Conclusions

In summary, in this study, we identified circRNA_101277 as a novel driver of CRC cell cisplatin resistance that functions by sequestering miR-370 and thereby positively regulating the expression of IL-6. Our data highlight the key roles of this circRNA_101277/miR-370/IL-6 axis in the context of CRC chemoresistance and suggest that this pathway may be a viable target for the diagnosis and treatment of this deadly cancer type.

## Figures and Tables

**Table 1:** Correlations between clinicopathological features and circRNA_101277 expression in 100 CRC tissue samples.

Clinicopathological features	circRNA_101277		*P* Value
	Low	High	
Age			0.722
≤60	28	27	
>60	22	23	
Gender			0.851
Male	26	27	
Female	24	23	
Tumor size			<0.05
<5 cm	37	11	
≧5 cm	13	39	
TNM stage			<0.05
I–II	38	15	
III–IV	12	35	
Metastasis			<0.05
No	32	14	
Yes	18	36	

## Data Availability

Due to the nature of this research, participants of this study did not agree for their data to be shared publicly, so supporting data are not available.
